# Rabconnectin-3α is required for the morphological maturation of GnRH neurons and kisspeptin responsiveness

**DOI:** 10.1038/srep42463

**Published:** 2017-02-17

**Authors:** Brooke K. Tata, Carole Harbulot, Zsolt Csaba, Stéphane Peineau, Sandrine Jacquier, Nicolas de Roux

**Affiliations:** 1 Univ Paris Diderot, Sorbonne Paris Cité, U1141, Inserm, F- 75019, Paris, France; 2 MRC Centre for Synaptic Plasticity; School of Physiology, Pharmacology, Neurosciences; University of Bristol, Bristol, UK; 3 AP-HP, Laboratoire de Biochimie. Hôpital Robert Debré, Paris, F-75019, France

## Abstract

A few hundred hypothalamic neurons form a complex network that controls reproduction in mammals by secreting gonadotropin-releasing hormone (GnRH). Timely postnatal changes in GnRH secretion are essential for pubertal onset. During the juvenile period, GnRH neurons undergo morphological remodeling, concomitantly achieving an increased responsiveness to kisspeptin, the main secretagogue of GnRH. However, the link between GnRH neuron activity and their morphology remains unknown. Here, we show that brain expression levels of *Dmxl2*, which encodes the vesicular protein rabconnectin-3α, determine the capacity of GnRH neurons to be activated by kisspeptin and estradiol. We also demonstrate that *Dmxl2* expression levels control the pruning of GnRH dendrites, highlighting an unexpected role for a vesicular protein in the maturation of GnRH neuronal network. This effect is mediated by rabconnectin-3α in neurons or glial cells afferent to GnRH neurons. The widespread expression of *Dmxl2* in several brain areas raises the intriguing hypothesis that rabconnectin-3α could be involved in the maturation of other neuronal populations.

GnRH neurons are integral members of a complex neuronal network within the hypothalamus that controls puberty onset and fertility^1^. Puberty is due to the reactivation of the hypothalamic-pituitary-gonadal (HPG) axis, involving a robust increase in GnRH pulsatile release to elicit luteinizing hormone (LH) and follicle-stimulating hormone (FSH) secretion from the anterior pituitary^2^,^3^. LH and FSH in turn stimulate gonadal sex steroids secretion and gametogenesis. Since 2003, kisspeptins (Kp) have emerged as major hypothalamic peptides, which control GnRH release (for a review see ref. 4). In fact, Kp act through the activation of a G-protein coupled receptor (KISS1R (GPR54)), which is expressed on the surface of GnRH neurons. Loss of function of GPR54 has been initially shown to cause GnRH deficiency^5^,^6^. Thereafter, Kp neurons were revealed to mediate the negative feedback of sex steroid hormones, as well as the positive feedback of the estradiol on the gonadotropic axis. The reactivation of the gonadotropic axis at puberty is associated with an increase of Kp signaling in GnRH neurons. Failure of the GnRH system to develop or function properly has been largely described and may be associated with complex syndromes characterized by lack of pubertal onset and neurodevelopmental defects^7^,^8^. Although mutations have been identified in several genes linked to these syndromes, the mechanism of the neurodevelopmental defect remains obscure.

We recently described a new complex neuronal disorder in humans associated with pubertal and fertility defects due to low expression of *DMXL2*^9^. Investigations in a *Dmxl2* deficient mouse line revealed that haploinsufficiency of *Dmxl2* in neurons (*nes::cre;Dmxl2*^*wt/loxp*^) causes infertility due to a partial GnRH deficiency^9^. *Dmxl2* encodes the vesicular protein rabconnectin-3α (rbcn3-α), which was first identified as a scaffolding protein and a protein partner of the GTPase protein Rab3A^10^,^11^, specifically Rab3-GTPase activating protein (Rab3-GAP) and Rab-3 Exchange Protein (Rab3-GEP). Rbcn3-α has also been shown important for cell-cell signaling and intracellular receptor trafficking^12^, as well as in the acidification of intracellular organelles by interacting with a subunit of the V-ATPase proton pump^13^,^14^.

We previously found that rbcn-3α is expressed in the organum vasculosum of the lamina terminalis (OVLT) and in the median eminence (ME)^9^, where GnRH neurons undergo extensive dendritic morphogenesis during the juvenile period of rodent life^15^. Rbcn-3α-immunoreactive vesicles were previously found expressed inside GnRH nerve terminals in the ME^9^, suggesting a possible role of rbcn-3α in GnRH release. Interestingly, GnRH neuronal dendrites possess spines, receive extensive synaptic inputs along their entire length^16^ and exhibit a striking degree of structural and functional plasticity over postnatal rodent development^15^. However, the link between GnRH neuron activity and their morphology remains unknown.

We previously reported that *nes::cre;Dmxl2*^*wt/loxp*^mice display a 30% loss of GnRH-immunoreactive neurons^9^. As such defect cannot fully explain the reproductive deficit observed in these mice, we suspected that an additional functional alteration might affect the GnRH neuronal network of *nes::cre;Dmxl2*^*wt/loxp*^ mice. As the use of the cre recombinase under the control of the nestin promoter results in the deletion of the critical exon in neuronal progenitors^17^, we thus sought to clarify whether low expression of rbcn3-α in neurons or glial cells may disturb the maturation and the activation of GnRH neurons, and what is the underlying mechanism leading to the reproductive deficit in these mice. As rbcn3-α is ubiquitously expressed in the brain and its function is vital after birth, we propose that the characterization of rbcn3-α function in the GnRH neuronal network will bring new insights on the understanding of neuronal maturation.

## Results

### *Dmxl2* low expression impedes the morphological changes of the GnRH neuronal dendritic tree at puberty

**Figure 1:**
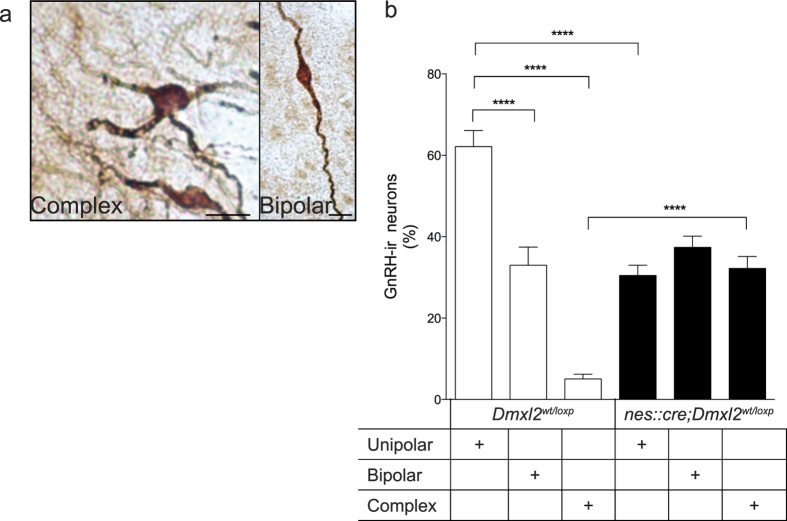
Dmxl2 neuronal deletion impairs the changes in GnRH neuronal morphology. (**a**) Photomicrographs representing complex/immature GnRH dendritic morphology in *nes::cre;Dmxl2*^*wt/loxp*^ mice and bipolar/mature GnRH dendritic morphology in *Dmxl2*^*wt/loxp*^ mice. Scale bar: 10-μm. (**b**) Quantification of the percentage of GnRH-immunoreactive dendritic morphologies in the rPOA in *Dmxl2*^*wt/loxp*^ and *nes::cre;Dmxl2*^*wt/loxp*^ mice (*n* = 5 animals per group, mean ± SEM, ****p < 0.0001).

Different studies have established that morphology, spine density, and topography of GnRH neurons are important for GnRH function^15^,^18^. In particular, GnRH neurons exhibit more complex/immature dendritic arborization during the juvenile period, which is then consolidated to a unipolar or bipolar (mature) morphology in adulthood^15^,^18^. The importance of GnRH dendritic morphological changes during puberty has yet to be studied. Thus, we first aimed at addressing whether ablation of *Dmxl2* in neurons or glial cells could interfere with the morphological maturation of GnRH neuronal dendrites during puberty. For that, we first analyzed GnRH dendritic morphology in the OVLT, located in the rostral preoptic area (rPOA) of adult *nes::cre;Dmxl2*^*wt/loxp*^mice, a region where the majority of GnRH cell bodies are located. We compared the GnRH dendritic morphologies between *nes::cre;Dmxl2*^*wt/loxp*^ adult mice and *Dmxl2*^*wt/loxp*^ mice. The percentages of GnRH unipolar, bipolar and complex neurons in the OVLT-rPOA in *Dmxl2*^*wt/loxp*^ mice were 62.1 ± 3.9%, 33.0 ± 4.4%, and 5.0 ± 1.1%, respectively (Fig. 1a,b). By contrast, *nes::cre;Dmxl2*^*wt/loxp*^ mice exhibited a blunted maturation of GnRH neuronal dendrites, showing a lower percentage of unipolar GnRH dendritic morphology (37.3 ± 2.7%), with a similar percentage of bipolar GnRH dendritic morphology (30.4 ± 2.5%), and significantly higher complex GnRH dendritic morphology (32.1 ± 2.9%) as compared to control mice (Fig. 1a,b).

### Kp-10 fails to induce LH secretion and GnRH neuron activation in *nes::cre;Dmxl2* ^*wt/loxp*^ male mice

**Figure 2:**
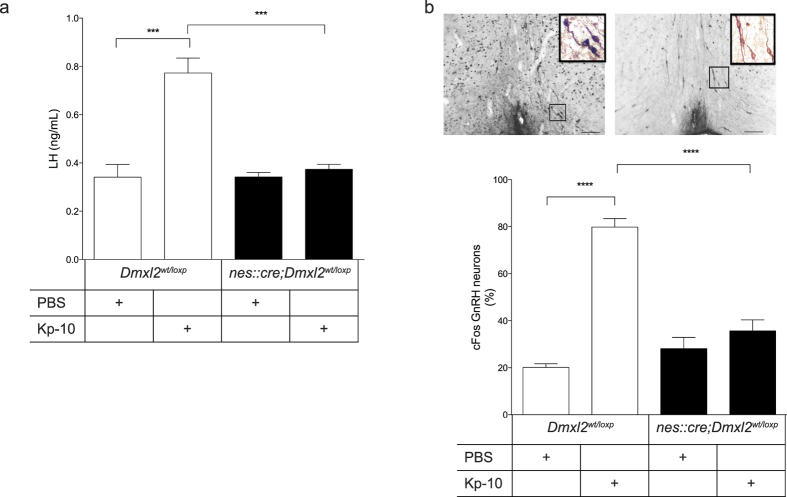
OVLT GnRH neurons in the rostral preoptic area do not respond to Kp-10 stimulation in *nes::cre;Dmxl2*^*wt/loxp*^ male mice. (**a**) Plasma LH levels (**b**) Photomicrograph depicts cFos positive GnRH neurons in Kp-10 treated *Dmxl2*^*wt/loxp*^ (left) and *nes::cre;Dmxl2*^*wt/loxp*^ (right) mice. Scale bar: 200-μm. Bottom graph depicts percentage of cFos positive GnRH neurons in Kp-10 and PBS treated *nes::cre;Dmxl2*^*wt/loxp*^ male mice compared to *Dmxl2*^*wt/loxp*^ males (*n* = 6 for each group, mean ± SEM, ****p < 0.0001). White bar: *nes::cre;Dmxl2*^*wt/loxp*^ male mice. Black bar: *nes::cre;Dmxl2*^*wt/loxp*^ male mice.

The abnormal GnRH dendritic morphologies in *nes::cre;Dmxl2*^*wt/loxp*^ mice suggested a defect in the functional maturation of GnRH neurons. To address this, we analyzed if Kp-10 intraperitoneal (i.p) injections were able to induce LH secretion in *nes::cre;Dmxl2*^*wt/loxp*^ male mice compared to *Dmxl2*^*wt/loxp*^ littermates. Indeed, Kp-10 induced a large increase in LH concentration in *Dmxl2*^*wt/loxp*^ male mice, whereas no rise in LH concentration was seen after *nes::cre;Dmxl2*^*wt/loxp*^ mice were subjected to Kp-10 (Fig. 2a). We next investigated if the absence of this response could be due to a failure of Kp-10 to induce GnRH neuron activation. To address this, analysis of cFos positive labeled rPOA GnRH neuronal nuclei was used as a read-out of Kp-10-induced GnRH neuron activation^19^. Injections of Kp-10 (i.p.) in *Dmxl2*^*wt/loxp*^ male mice induced an increase in the percentage of rPOA cFos-GnRH positive nuclei as compared to PBS-treated *Dmxl2*^*wt/loxp*^ male mice (Fig. 2b). By contrast, the percentage of dually labeled cFos-GnRH neurons in Kp-10-treated *nes::cre;Dmxl2*^*wt/loxp*^ mice was not different to that of PBS-treated *nes::cre;Dmxl2*^*wt/loxp*^ male nor PBS-treated *Dmxl2*^*wt/loxp*^ mice (Fig. 2b).

### GnRH neurons in *nes::cre;Dmxl2* ^*wt/loxp*^ female mice do not respond to the E_2_ positive feedback paradigm

**Figure 3:**
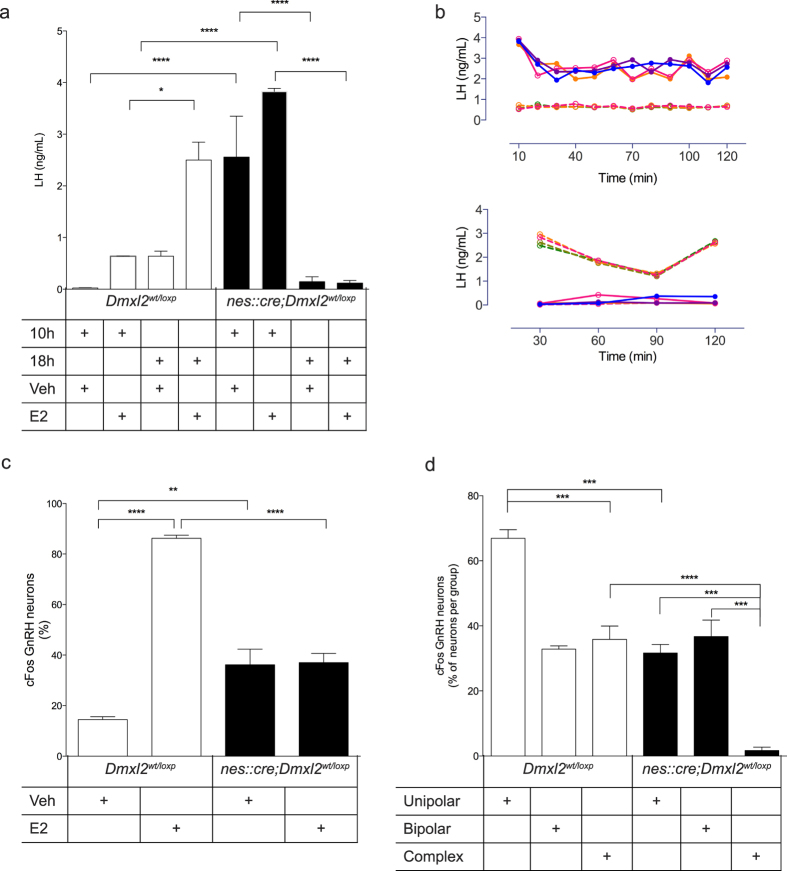
GnRH neurons in OVX *nes::cre;Dmxl2*^*wt/loxp*^ female mice are unresponsive to E_2_-positive feedback. (**a**) Plasma LH levels measured at 10:00 h and 18:00 h in both E_2_- and Veh-treated *nes::cre;Dmxl2*^*wt/loxp*^ and *Dmxl2*^*wt/loxp*^ ovariectomized (OVX) female mice (*n* = 5 for each group and time point, mean ± SEM, **p < 0.01, ****p < 0.0001). (**b**) Top graph depicts the LH levels in a pulsatile manner during 10-min intervals for 2-hours in OVX + E2 *Dmxl2*^*wt/loxp*^ (*n* = 4) and OVX + E2 *nes::cre;Dmxl2*^*wt/loxp*^ mice (*n* = 4) mice between 10:00 h and 12:00 h. Bottom graph depicts LH concentrations over 30-min intervals for 2-hours between 18:00 h and 20:00 h in OVX + E2 *Dmxl2*^*wt/loxp*^ (*n* = 5) and OVX + E2 *nes::cre;Dmxl2*^*wt/loxp*^ mice (*n* = 4). Dashed lines; *Dmxl2*^*wt/loxp*^ mice. Solid line; *nes::cre;Dmxl2*^*wt/loxp*^ mice. (**c**) cFos positive rPOA GnRH-ir neurons in OVX + E_2−_ and OVX + Veh-treated *nes::cre;Dmxl2*^*wt/loxp*^ and *Dmxl2*^*wt/loxp*^ adult female mice. Percentage of the total number of GnRH neurons (**d**) cFos positive GnRH neurons related to the GnRH neuron morphology in Kp-10-treated male (*n* = 4) and E_2_-treated female mice at 18 h (*n* = 4) (mean ± SEM, ***p < 0.001, ****p < 0.0001). Percentage of cFos-GnRH-ir neurons to the total number of counted GnRH neuron in each group. The numbers of unipolar, bipolar and complex cFos-positive GnRH neurons counted in each subgroup were 74, 60, 8 in *Dmxl2*^*wt/loxp*^ mice and 16, 12, 2 in *nes::cre;Dmxl2*^*wt/loxp*^ mice, respectively. White bar: *nes::cre;Dmxl2*^*wt/loxp*^ mice. Black bar: *nes::cre;Dmxl2*^*wt/loxp*^ mice.

We previously reported that *nes::cre;Dmxl2*^*wt/loxp*^ female mice exhibit a normal development of antral follicles but lower number of corporal lutea, which was associated with infertility^9^. The infertility in female *nes::cre;Dmxl2*^*wt/loxp*^ mice could therefore be related to the inability of GnRH neurons to respond to the Estradiol (E_2_)-induced positive feedback. In rodents, high E_2_ leads to positive feedback, which is mediated by increased Kp-10 signaling in GnRH neurons, leading to a GnRH/LH surge at the time of lights out on the day of proestrus^20^,^21^. Ovariectomized (OVX)-*Dmxl2*^*wt/loxp*^ female mice implanted with low E_2_ capsule for 1-week exhibited higher LH values at 18:00 h (LH surge) when given a bolus of E_2_ as compared to vehicle (Veh)-treated OVX- *Dmxl2*^*wt/loxp*^ females (Fig. 3a). However, *nes::cre;Dmxl2*^*wt/loxp*^ mice could not elicit a LH surge at the time of lights out despite the hormonal regimen (Fig. 3a). Indeed, LH levels in tail-tip sampling over two hours between 18:00–20:00 h remained very low, showing no sign of an LH peak or surge, whereas OVX + E_2_ treated *Dmxl2*^*wt/loxp*^ exhibited a LH surge (Fig. 3b), Unexpectedly, *nes::cre;Dmxl2*^*wt/loxp*^ mice exhibited significantly higher LH values at 10:00 h as compared to 18:00 h when given a bolus of E_2_, and the same results were found when they were given vehicle treatment (Fig. 3a,b).

To assess proper GnRH neuron activation at the time of the LH surge, the percentage of cFos-GnRH neurons was analyzed at 18:00 h in OVX + E_2_ female mice. A significant increase in cFos-GnRH positively labeled GnRH neurons was observed in E_2_-treated *Dmxl2*^*wt/loxp*^ female mice as compared to Veh-treated *Dmxl2*^*wt/loxp*^ mice (Fig. 3c). By contrast, E_2_-treated *nes::cre;Dmxl2*^*wt/loxp*^female mice exhibited the same percentage of cFos-GnRH positively labeled GnRH neurons as Veh-treated *nes::cre;Dmxl2*^*wt/loxp*^ females (Fig. 3c). To note, Veh-treated *nes::cre;Dmxl2*^*wt/loxp*^ female mice exhibited a significant higher cFos-GnRH positive staining at 18:00 h when compared to Veh-treated *Dmxl2*^*wt/loxp*^ females. Collectively, these data show that *nes::cre;Dmxl2*^*wt/loxp*^ female mice do not display a proper positive feedback response to E_2_, which is compounded by an abnormal timing of the LH surge in the morning. In addition, a constitutive neuronal activation was observed at 18:00 h in both Veh- and E_2_-treated *nes::cre;Dmxl2^wt:loxp^* animals.

We next assessed whether the defect in GnRH-neuronal activation in *nes::cre;Dmxl2*^*wt/loxp*^ mice was potentially associated to their defect in GnRH morphological maturation. To test this, cFos-GnRH expression was quantified per group of GnRH neurons based on their morphology in both Kp-10 treated and OVX + E_2_ treated *Dmxl2*^*wt/loxp*^ and *nes::cre;Dmxl2*^*wt/loxp*^ male and female mice. Unipolar GnRH neurons displayed a higher cFos staining than bipolar neurons in wild type mice (Fig. 3d). Despite the fact that complex/immature GnRH neurons responded to Kp-10 and E_2_ in *Dmxl2*^*wt/loxp*^ mice, it should be noted that there were extremely few immature GnRH neurons in *Dmxl2*^*wt/loxp*^mice. By contrast, *nes::cre;Dmxl2*^*wt/loxp*^ mice exhibited significantly more complex/immature GnRH neurons with almost no cFos-GnRH positive labeling (Fig. 3d). With this, *nes::cre;Dmxl2*^*wt/loxp*^ mice displayed significantly less cFos-GnRH positive labeling in unipolar and complex GnRH neurons, whereas no change was observed in bipolar neurons (Fig. 3d). In summary, the number of complex GnRH neurons was higher in *nes::cre;Dmxl2*^*wt/loxp*^ mice and their capacity to be activated was almost completely abolished. In contrast to unipolar or complex neurons, bipolar neurons appeared to be insensitive to low expression of *Dmxl2* in neuronal progenitor-derived cells.

### Kisspeptin expression in the antero-ventral periventricular nucleus (AVPV/PeN) is disturbed in *nes::cre;Dmxl2* ^*wt/loxp*^ mice

**Figure 4:**
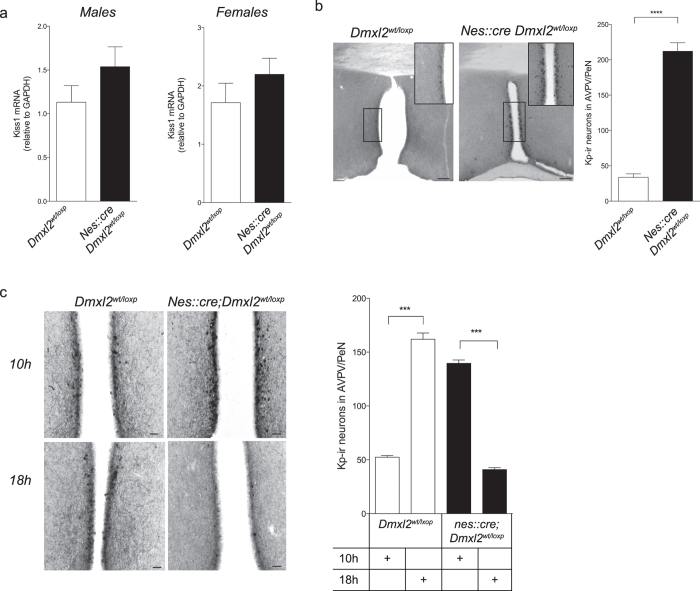
Kisspeptin expression is altered in *nes::cre;Dmxl2*^*wt/loxp*^ mice. **(a)** Relative expression of hypothalamic *Kiss1* mRNA in *Dmxl2*^*wt/loxp*^ (males: *n* = 7; females: n = 5) and *nes::cre*;*Dmxl2*^*wt/loxp*^ adult mice (males: *n* = 10; females: n = 8) (*p* > 0.05). (**b**) Left: Photomicrograph depicting Kp-ir neurons in the AVPV/PeN in one adult *Dmxl2*^*wt/loxp*^ (top) and *nes::cre;Dmxl2*^*wt/loxp*^ (bottom) male mice. Scale bars 300-μM. Quantification of AVPV/PeN Kp-ir neurons in *Dmxl2*^*wt/loxp*^ (*n* = 5) and *nes::cre;Dmxl2*^*wt/loxp*^ male mice (*n* = 5; Mean ± SEM,****p < *0.001*). (**c**) Photomicrograph depicts Kp-ir staining in the AVPV/PeN in OVX + E2 treated *Dmxl2*^*wt/loxp*^ (left) and *nes::cre;Dmxl2*^*wt/loxp*^ (right) female mice at 10:00 h and 18:00 h. Scale Bar 10-μM. (**d**) Quantification of AVPV/PeN Kp-ir neurons in OVX + E2 treated *Dmxl2*^*wt/loxp*^ (*n* = 4–5) and *nes::cre;Dmxl2*^*wt/loxp*^ (*n* = 4–5) female mice at 10:00 h and 18:00 h (Mean ± SEM, ***p < *0.001*). White bar: *nes::cre;Dmxl2*^*wt/loxp*^ mice. Black bar: *nes::cre;Dmxl2*^*wt/loxp*^ mice.

To analyze the consequences of *Dmxl2* neuronal haploinsufficiency on kisspeptin neurons, we first quantified *Kiss1* mRNA by quantitative PCR (qRT-PCR) from total RNA extracted from hypothalamus of *nes::cre;Dmxl2*^*wt/loxp*^ mice. This analysis was performed in males and in diestrus females at PND 60. In both sexes, we did not observe any difference of the relative levels of hypothalamic *Kiss1* mRNA to *GAPDH* between *Dmxl2*^*wt/loxp*^ and *nes::cre;Dmxl2*^*wt/loxp*^ mice (Fig. 4a). This first result indicated that the gonadotropic deficiency observed in *nes::cre;Dmxl2*^*wt/loxp*^ mice was not due to a dramatic change of *Kiss1* expression in the hypothalamus. However, our analysis did not take into account the fact that Kp neurons in the anteroventro/periventricular (AVPV/PeN) nucleus are positively controlled by estradiol in female mice, whereas in the arcuate nucleus, estradiol acts as a negative regulator of *Kiss1* expression in both sexes. It was therefore necessary to assess Kp expression by another approach which differentiated Kp expression in the AVPV/PeN from the arcuate nucleus. To do this, we quantified the total number of Kp-ir neurons in the AVPV/PeN. In *nes::cre;Dmxl2*^*wt/loxp*^ males, we observed significantly higher number of AVPV/PeN Kp-ir neurons as compared to control males (Fig. 4b). In female mice, results were more complex. As expected, OVX + E2 treated *Dmxl2*^*wt/loxp*^ females displayed higher number of Kp-ir neurons in the AVPV/PeN at 18:00 h when compared to 10:00 h (Fig. 4c). By contrast, there were significantly more AVPV/PeN Kp-ir neurons in OVX + E2-treated *nes::cre;Dmxl2*^*wt/loxp*^ mice at 10:00 h as compared to 18:00 h (Fig. 4c). This staining corresponded to the inversed LH levels (See Fig. 3a,b). To note, Kp-ir staining in the arcuate nucleus was similar between wild type and *nes::cre;Dmxl2*^*wt/loxp*^ female mice in diestrus (data not shown). The consequences of low expression of rbcn3α in the brain thus differs between sexes. In *nes::cre;Dmxl2*^*wt/loxp*^ male mice, GnRH neurons exhibit a resistance to Kp associated with an increase of Kp expression in the AVPV/PeN. In *nes::cre;Dmxl2*^*wt/loxp*^ female mice, Kp expression analysis suggests a defect in the control of its diurnal expression in AVPV/PeN.

### Lack of *Dmxl2* expression in GnRH neurons does not affect their functional maturation

**Figure 5:**
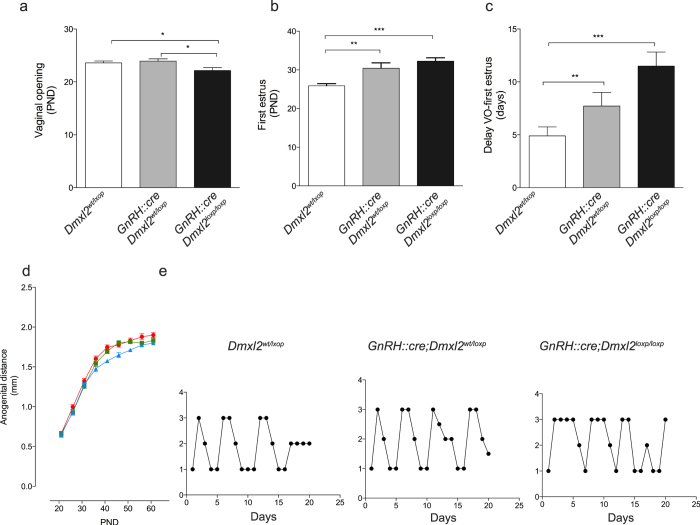
GnRH::cre;Dmxl2 knock-out mice display a normal reproductive phenotype. Parameters of pubertal onset in female *Dmxl2*^*loxp/loxp*^(*n* = 20), *GnRH::cre;Dmxl2*^*wt/loxp*^(*n* = 18), and *GnRH::cre;Dmxl2*^*loxp/loxp*^ (*n* = 7) mice. **(a)** Age of VO and (**b**) age of first estrus and (**c**) time between VO and first estrus. (**d**) Measurement of the anogenital distance (AGD, mm) in *Dmxl2*^*loxp/loxp*^(blue, *n* = 5), *GnRH::cre;Dmxl2*^*wt/loxp*^(green, *n* = 6), and *GnRH::cre;Dmxl2*^*loxp/loxp*^ (red, n = 8) male mice over PND 20–60. Asterisks represent significant differences (mean ± SEM *p < 0.05, **p < 0.005, ***p < 0.0001). (**e**) Representative graphs depicting estrous cyclicity over 21 days (5-cycles) in *Dmxl2*^*loxp/loxp*^(*n* = 20), *GnRH::cre;Dmxl2*^*wt/loxp*^(*n* = 18), and *GnRH::cre;Dmxl2*^*loxp/loxp*^ (*n* = 7) female adult mice. Numerical values represent stage of the cycle: 3 (Proestrus), 2 (Estrus), 1 (Metestrus/Diestrus).

**Figure 6:**
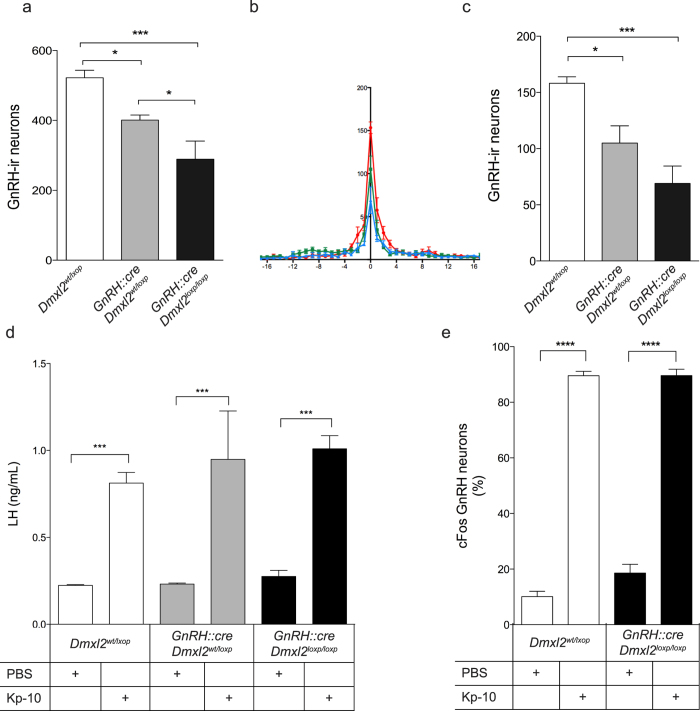
*GnRH::cre;Dmxl2* knock-out mice exhibit GnRH neuronal loss yet a normal GnRH neuron response to Kp-10. (**a–c**) Quantification and distribution of GnRH-ir neurons in *Dmxl2*^l*oxp/loxp*^, GnRH::cre;*Dmxl2*^*wt/loxp*^, and GnRH::cre;Dmxl2^loxp/loxp^ mice (*n* = 5 in all genotypes). (**a**) Total number of GnRH-ir neurons in the hypothalamus and (**b**) Rostral to caudal distribution of GnRH-ir neurons, where OVLT is marked numerically as 0, with rostral GnRH-ir neurons in negative numerical values and caudal GnRH-ir neurons in positive numerical values. Dmxl2^loxp/loxp^ (blue), GnRH::cre;*Dmxl2*^*wt/loxp*^ (green), GnRH::cre;Dmxl2^loxp/loxp^ (red) (**c**) Total number of GnRH-ir neurons in the OVLT. (**d**) Plasma LH concentration in Kp-10 and PBS-treated Dmxl2^loxp/loxp^, GnRH::cre;*Dmxl2*^*wt/loxp*^, and GnRH::cre;Dmxl2^loxp/loxp^ mice (*n* = 5 in all groups and genotypes). (**e**) cFos-GnRH neurons in both Kp-10 and PBS-treated Dmxl2^loxp/loxp^ and GnRH::cre;Dmxl2^loxp/loxp^ male mice (*n* = 5 in all groups and genotypes) (mean ± SEM, *p < 0.05, **p < 0.01, ***p < 0.001). White bar: Dmxl2^loxp/loxp^. Gray bar: GnRH::cre;*Dmxl2*^*wt/loxp*^, Black bar: GnRH::cre;Dmxl2^loxp/loxp^.

As rbcn3-α is expressed in GnRH neurons^9^, the functional defect observed in *nes::cre;Dmxl2*^*wt/loxp*^ could be attributable to deletion of *Dmxl2* in GnRH neurons alone, or cumulative defects in both afferent and GnRH neurons as well as in afferent neurons or glial cells only. To test this hypothesis, we created and analyzed another mouse model with ablated *Dmxl2* in GnRH neurons. GnRH*::cre;Dmxl2*^*wt/loxp*^ mice exhibited a slight delay in pubertal onset and *GnRH::cre;Dmxl2*^*loxp/loxp*^ mice showed a mild reproductive phenotype with a delayed first ovulation but a normal ovarian cyclicity in adult female mice, and a normal ano-genital distance (AGD) in male mice (Fig. 5a–e). To assess if there was a defect in the number of GnRH neurons, we analyzed the number and the distribution of GnRH neurons in the three genotypes. *GnRH::cre;Dmxl2*^*wt/loxp*^ and *GnRH::cre;Dmxl2*^*loxp/loxp*^ mice had significantly less GnRH-ir neurons as compared to *Dmxl2*^*loxp/loxp*^ mice (Fig. 6a). This loss of GnRH-ir neurons was most significant in the OVLT in both *GnRH::cre;Dmxl2*^*wt/loxp*^ and *GnRH::cre;Dmxl2*^*loxp/loxp*^ mice as compared to wild type mice (Fig. 6b,c). Because *nes::cre;Dmxl2*^*wt/loxp*^ mice harbored a more severe reproductive phenotype to that of *GnRH::cre;Dmxl2*^*wt/loxp*^ mice, yet a similar GnRH neuronal loss to that of *GnRH::cre;Dmxl2*^*wt/loxp*^ mice and *GnRH::cre;Dmxl2*^*loxp/loxp*^, we questioned what separated the phenotype in *GnRH::cre;Dmxl2* knock out mice from that of *nes::cre;Dmxl2*^*wt/loxp*^ mice. To test this, we subjected *Dmxl2*^*loxp/loxp*^, GnRH*::cre;Dmxl2*^*wt/loxp*^ and *GnRH::cre;Dmxl2*^*loxp/loxp*^ mice to Kp-10 stimulation. A Kp-10-induced LH increase was observed in mice of all genotypes, (Fig. 6d). Confirming proper GnRH neuron activation, we found that the number of cFos positive GnRH neurons in Kp-10-treated *GnRH::cre;Dmxl2*^*loxp/loxp*^ mice did not differ from that of Kp-10-treated *Dmxl2*^*loxp/loxp*^ mice (Fig. 6e). The cumulative reproductive deficit in *nes::cre;Dmxl2*^*wt/loxp*^mice thus extends beyond the GnRH neuron deficit alone.

## Discussion

Herein, we found that *Dmxl2* deficiency in mouse brain reduces the physiological transition of immature GnRH neurons toward mature neurons along with a reduced GnRH immunoreactivity within the OVLT and an absence of GnRH neuronal responsiveness to Kp-10 in males. This resistance to Kp-10 was associated to an increase of Kp expression in the AVPV/PeN. By contrast to male mice, *nes::cre;Dmxl2*^*wt/loxp*^ female mice did not develop a kisspeptin resistance but rather an abnormal control of Kp expression during the day. Although we observed a decrease of GnRH-ir neurons in mice in which *Dmxl2* was deleted solely in GnRH neurons, the reproductive phenotype observed was mild and did not yield the functional defect as to which we observed in *nes::cre;Dmxl2*^*wt/loxp*^ mice. These results reveal the critical role of the expression level of rbcn-3α in the brain for the post-natal homeostasis of the GnRH neuronal network.

The analysis of *GnRH::cre;Dmxl2* knock-out mice highlights that the cumulative functional defect observed in *nes::cre;Dmxl2*^*wt/loxp*^ mice is most likely due to the extrinsic role of rbcn3-α in afferent neurons or in glial cells, with a less important intrinsic role in GnRH neurons. Indeed, cre-recombinase under the control of the nestin promoter leads to a deletion of the critical exon at the stage of neuronal progenitors which can be further differentiated in neurons or glial cells^17^. In addition to the crucial role of several hypothalamic neurons, the contribution of glial cells in the control of GnRH neurons is well known^2^. Glial cells secret small molecules that directly activate GnRH neurons^2^. Glial cells also participate to the control of the GnRH release at the neurohemmal junction in the ME upon the control of nitric oxide produced by endothelial cells^22^. As rbcn3-α is expressed in tanycytes in the ME^9^, a dysfunction in the structural plasticity of tanycytes could perturb the control of GnRH release and thus could contribute to the phenotype in *nes::cre;Dmxl2*^*wt/loxp*^ female mice. However, we suspect that this effect likely plays a minor role in the phenotype of these mice when compared to the abnormal maturation of GnRH neuronal dendrites with the disorganized control of Kp expression.

GnRH neurons respond to endogenous Kp concomitantly with their morphological remodeling^15^, however, the functional link between these two events has not been previously explored. To date, complex GnRH dendritic morphology was reported in only one mouse model exhibiting delayed puberty and fertility defects^23^, yet the functional relevance of the immature GnRH morphologies was not explored^23^. In our study, we found an increase of complex/immature GnRH dendritic morphology in the OVLT in *nes::cre;Dmxl2*^*wt/loxp*^ mice, associated with a decrease of unipolar morphology, and no changes in the bipolar GnRH dendritic morphology. These data indicate that the balance between the stabilized-to-destabilized GnRH dendritic morphologies is disturbed when the expression of *Dmxl2* is low in the brain. Complex/immature GnRH dendritic morphology in *nes::cre;Dmxl2*^*wt/loxp*^ mice failed to exhibit cFos activation after Kp-10 and/or E_2_ treatment, whereas mature unipolar GnRH neurons, albeit at a lesser level, still exhibited significantly higher cFos activation than complex neurons. Unipolar and bipolar GnRH neurons are thus more prone to be stimulated by Kp-10 and E_2_ than complex neurons in *nes::cre;Dmxl2*^*wt/loxp*^ mice. These results pinpoint that the dendritic pruning of GnRH neurons during the juvenile period could participate to the pubertal increase of GnRH neuron responsiveness to Kp and E_2_. It could also be a concomitant event without any functional relationship to the increase Kp effect on GnRH neurons at puberty.

Surprisingly, LH blood concentrations were increased at 10:00 h in E_2_-treated *nes::cre;Dmxl2*^*wt/loxp*^ mice. The correlation between LH concentrations with Kp expression in the AVPV/PeN in OVX + E_2_ *nes::cre;Dmxl2*^*wt/loxp*^ mice at different times of the day, implies that GnRH neurons likely responded to endogenous Kp in the morning (10:00 h) instead at night of proestrus at lights out (18:00 h). In *nes::cre;Dmxl2*^*wt/loxp*^ adult males, the relatively mild effect that *Dmxl2* haploinsufficiency had on testis weight contrasts with the complete resistance of GnRH neurons to Kp-10. In fact, *Kiss1R* knock out mice exhibit very small testes in adulthood^5^. Further studies are necessary to delineate the age at which GnRH neurons become resistant to Kp-10 in *nes::cre;Dmxl2*^*wt/loxp*^ male mice. We suspect the major difference of Kp signaling between sexes in *nes::cre;Dmxl2*^*wt/loxp*^ mice is related to the sexual dimorphism of the GnRH neuronal network^24^.

High LH levels were also observed in Veh-treated *nes::cre;Dmxl2*^*wt/loxp*^ mice indicating that this increase in LH is likely E_2_ independent. The abnormal high expression of Kp in the AVPV/PeN at 10:00 h *nes::cre;Dmxl2*^*wt/loxp*^ female mice revealed an advanced phase of Kp expression. The circadian timing of the GnRH neuronal network is under the control of neurons of the suprachiasmatic nucleus (SCN)^25^. As rbcn3-α is expressed in the ventral part of the SCN^9^, an abnormal control of the GnRH neuronal network by the SCN may be one explanation for the advanced phase of the LH release. Arginine vasopressin (AVP) and vasoactive intestinal peptide (VIP) are both expressed by SCN neurons. AVP and VIP neurons were shown to contact Kp neurons and GnRH neurons, respectively^26^. AVP exerts daily signals onto Kp neurons, of which is highly dependent on circulating levels of estradiol, but not on the time of the day^27^. VIP neurons has also been shown to participate to the GnRH/LH surge but it is probably less critical than AVP for the timing of the surge during the late afternoon of proestrus^28^. In addition, Kp neurons were shown to express clock genes and to display an intrinsic circadian oscillator upon the control of estradiol^29^. Further studies will delineate whether the abnormal daily expression of Kp in the AVPV/PeN in *nes::cre;Dmxl2*^*wt/loxp*^ mice is related to an abnormal control of the GnRH neuronal network by AVP/VIP neurons and/or to an alteration of the intrinsic circadian oscillator of Kp neurons.

Changes in synaptic inputs and cell-cell signaling onto GnRH neurons contribute to the GnRH neuronal plasticity^30^,^31^,^32^,^33^. These synaptic inputs can change afferent neuronal activity as well as retraction or apposition of synapses on GnRH neuron perykaria^34^. A defect in the synaptic plasticity in GnRH neurons in *nes::cre;Dmxl2*^*wt/loxp*^ mice could explain in part the altered response to Kp-10 and E_2_ stimulation^35^. The biochemical function of rbcn3-α supports the hypothesis of a synaptic defect when *Dmxl2* expression is low in neurons. Indeed, rbcn-3α participates in the acidification of intracellular vesicles through the control of the V-ATPase activity^13^,^14^,^36^, where acidification is necessary for an optimal synaptic function. Many other cellular processes such as protein-processing and receptor-mediated endocytosis may also be disturbed^37^. GnRH neurons receive inputs from GABAergic and glutamatergic neurons^34^, of which both express estrogen receptor 1 (ESR1). The homeostasis of the GnRH neuronal network is highly disturbed in ESR1-deleted glutamatergic neuron mice^38^. In some-way, the phenotype observed in *nes::cre;Dmxl2*^*wt/loxp*^ female mice is similar to the disrupted homeostasis of the GnRH neuronal network observed in ESR1-deleted glutamatergic neuron mice. The increase of the basal cFos staining in GnRH neurons in *nes::cre;Dmxl2*^*wt/loxp*^ female mice as opposed to wild type females implies there could be an imbalance between excitatory and inhibitory synaptic inputs on GnRH neurons. This could be also due to the fact that there are less mature GnRH neurons and thus a compensatory mechanism is trying to override the poorly functional immature GnRH neurons. These results suggest a functional link between estrogen receptor signaling pathways, glutamatergic inputs on the GnRH neuronal network and the control of the V-ATPase activity.

Altogether, these results revealed the crucial role of the expression level of *Dmxl2* in the brain for the functional and morphological maturation of GnRH neurons by afferent neurons or glial cells in mice. We also revealed the role of a vesicular protein on the timing of the LH surge upon the control of estradiol. This is a new mechanism of gonadotropic deficiency in mice, which is likely similar in *DMXL2* mutated patients. This study opens new avenues for a better understanding of the mechanisms leading to GnRH neuron dysfunction.

## Methods

### Animals

*nes::cre;Dmxl2* and *GnRH::cre* mice have been previously characterized^9^,^39^. To obtain *GnRH::cre;Dmxl2*^*loxp/loxp*^ mice, we crossed *Dmxl2*^*loxp/loxp*^ mice with *GnRH::cre* transgenic mice. Both *nes::cre;Dmxl2* and *GnRH::cre;Dmxl2* lines were weaned at post-natal day (PND) 21 and tail biopsies were harvested for genotyping. *GnRH::cre;Dmxl2* female mice were checked for Vaginal Opening (VO) starting at PND 21. Upon VO, females were cycled for estrous cyclicity for 21 days to obtain 5 cycles. Anogenital Distance (AGD) was measured in male *GnRH::cre;Dmxl2* mice starting at PND 21 until PND 60. Animals were housed 4–5 per cage on a 12 h light: 12 h dark cycle (lights on 6am, lights off 18 h).

Animal use was in compliance with Inserm guidelines for the care and use of laboratory animals in accordance with Paris Diderot University and Inserm. Mammalian research was approved by the Institutional Ethics Committees of Care and Use of Experimental Animals by Inserm and Paris Diderot University with the ethical approval number 2012-15-676-0099.

### Perfusion

Animals were given an overdose of pentobarbital (3 mg/100-μL) and intracardially perfused with 4% paraformaldehyde (PFA) in 0.1 M Phosphate Buffer (PB) (pH 7.6). Brains were post-fixed overnight at 4 °C, placed in 30% Sucrose/TBS (pH 7.6), frozen in 99% isopentane and kept at −80 °C until sectioning.

### Immunohistochemistry

For each experiment, five adult males (>PND 60) from each genotype, tissues were sectioned (Leica) in 40-μm serial coronal sections from the the Medial Septum (MS) to the rostral preoptic Area (rPOA).

#### Chromagen labeling

Endogenous peroxidase activity was inhibited with 40% methanol, 1% H_2_0_2,_ and 0.05 M TBS (pH 7.6). Sections were rinsed with TBS1x and blocked in an incubation solution (ICS) containing 10% normal goat serum, 0.25% BSA in 0.05 M TBS and 0.3% Triton 100-X, pH 7.6 for 1-hour at RT. Sections were incubated in ICS containing 10% NGS in 1/15000 guinea-pig polyclonal GnRH antibody (generous gift from Dr. Greg Anderson, Table S1) for 72-hrs at 4 °C for GnRH neuron immunoreactivity, or in 1/5000 rabbit polyclonal anti-Kisspeptin-10 antibody (AC564, generous gift from Dr. Alain Caraty, Table S1). After rinses, sections were incubated in biotinylated goat anti-guinea pig antibody or biotinylated donkey anti-rabbit antibody (1/200; Vector Laboratories) for 90-mins at RT, washed and incubated with avidin-peroxidase (1/200) in ICS for 90 mins at RT (Vectastain ABC Kit, Vector Laboratories). GnRH and Kp-10 immunoreactivity was revealed using 0.05% diaminobenzadine (DAB) (Sigma Aldrich) with 0.01% H_2_0_2_ in TBS 0.05 M (pH 7.6) for 15 mins at RT, then rinsed and coverslipped for analysis.

*Dual chromagen labeling* was carried out using the same method above with an addition step of using the glucose-oxidase/NiDAB method. Briefly, sections were incubated in rabbit polyclonal cFos primary antibody (1/5000; SC-52, Santa Cruz Biotechnology, Santa Cruz, CA), and 1/15000 guinea-pig polyclonal GnRH antibody (GA04; a generous gift from Dr. Greg Anderson, Table S1) in ICS solution at 4 °C for 48-hrs. After rinses, sections were incubated in biotinylated anti-rabbit (1/200; Vector Laboratories) for 90-min at RT, rinsed, and reacted with glucose oxidase and NiDAB for 5-mins at RT. Sections were then incubated in 10% NGS with polyclonal goat anti-guinea, (1/200; Vector Laboratories) for 90-mins at RT and reacted with 0.05% DAB (Sigma-Aldrich) in 0.01% H_2_0_2_ and 0.05 M TBS1x for 15-mins, rinsed and coverslipped for analysis.

### Quantification of GnRH neurons in *GnRH::Cre;Dmxl2* mice

Five adult males and females (>PN day 60) from each genotype were perfused and brains were sectioned coronally (45-uM) on a sliding microtome. Sections were processed for GnRH chromagen labeling immunohistochemistry (see above). Sections were mounted in rostral to caudal order from the Medial Septum (MS) to the Arcuate Nucleus (ARN). To obtain the global distribution of GnRH neurons, sections before the OVLT (rostral) were labeled with negative numbers and sections after the OVLT (caudal) were considered positive numbers. The OVLT was labeled as zero. GnRH neurons were quantified in order and plotted in overall distribution from the diagonal band of broca to the median eminence, as previously reported^9^.

### Quantification of dually labeled GnRH-cfos positive neurons in the OVLT-rPOA

To quantify cFos positive GnRH neurons, GnRH neurons were separated into two populations (OVLT and rPOA) where the distribution and number of dually-labeled cFos-GnRH neuronal nuclei were counted in the regions of the Franklin and Paxinos brain atlas plates 22–24, 25–27, and 28–31, respectively (Franklin and Paxinos, 1997).

### Quantification of GnRH dendritic morphologies

Three coronal sections containing GnRH-ir neurons from the MS to the rPOA were viewed on a Nikon brightfield microscope and morphologies were scored, as previously described^15^. Briefly, GnRH-ir neuronal morphologies were quantified based on three criteria: unipolar (mature; one dendrite directly off the GnRH soma), bipolar (mature; two dendrites directly off the GnRH soma), or complex (immature; three or more dendritic processes directly off of the GnRH soma)^15^. Values of quantified GnRH dendritic morphologies were expressed as the percentage of the total GnRH neuron population analyzed.

### *In vivo* studies

#### Kisspeptin-10 (Kp-10) Injections

Five adult male *nes::cre;Dmxl2*^*wt/loxp*^ and *GnRH::cre;Dmxl2*^*wt/loxp,*^ *GnRH::cre;Dmxl2*^*loxp*/loxp^, and *Dmxl2*^*loxp/loxp*^ mice were given intraperitoneal (i.p.) injections of 100-μl of 1-nmol Kp-10 (Sigma Aldrich) or PBS (control)^40^. 10-mins after injections, 4-μl of tail-tip blood was collected in duplicates. Mean LH levels were pooled through tail-tip sampling 10-mins after Kp-10 injections where tail tip blood was harvested for 2-hrs. LH levels were analyzed using an ELISA Luteinizing Hormone (LH) Sandwich Assay, as previously described^41^; *See ELISA LH Sandwich Assay*). For quantification of cFos labeling, 2-hours after i.p. injection of Kp-10 or PBS (control), male mice were perfused, as described above, and brains were prepared for immunolabeling (see *Immunohistochemistry*).

#### Positive Feedback Paradigm and Evaluation of E_2_-mediated positive feedback effects on LH levels

Adult *nes::cre;Dmxl2*^*wt/loxp*^ and *Dmxl2*^wt/loxp^ female mice, housed 4–5 per cage on a 12 h light (6am): 12 h dark (6 pm) were bilaterally ovariectomized (OVX) (Day 0) and implanted with 1-cm of low-dose E_2_ silastic capsules containing 17-β-estradiol (1 μg/20 g) (Sigma Aldrich; inner diameter 0.10 cm, external diameter 0.212 cm; Dow Corning, MI), and Silastic medical-grade adhesive (0.1 mg/mL; Dow Corning), as previously reported^21^. Six days after low-E_2_ treatment, OVX female mice were subjected to subcutaneous injections of either 1 μg/20 g estradiol benzoate (E_2_) mixed in sesame oil (100 μl) or sesame oil alone (Veh) as a control at (9:00 h). To measure LH pulsatility and to obtain LH pulsatility and the LH surge at the time of lights out (18h00), tail-tip blood samples were harvested every 10 mins between 10:00 h and 12:00 h or between 18:00–20:00 h the day after E_2_ and Veh treatment (See *LH Elisa*). To analyze cFos activation of GnRH neurons as well as AVPV Kp-10 immunoreactivity at different time points, we generated another group of adult OVX + E2 *Dmxl2*^*loxp/wt*^ and *nes::cre; Dmxl2*^*loxp/wt*^ females. After one week under hormonal regimen and 36-hrs after Estradiol Benzoate injection or Vehicle (Veh) treatment, animals were perfused for cFos analysis and for Kp immunoreactivity in AVPV/PeN (See *Immunohistochemistry*). To quantify Kp-ir neurons, three AVPV/PeN sections from each animal and treatment were quantified for the average number of Kp-ir neurons.

### Image Analysis

Light microscopy image acquisition was performed using an Zeiss LSM710 Apoptome. For Chromagen labeling for GnRH, cFos, and kisspeptin neurons, images were acquired using Axiovision software using both 10x and 20x Plan Neofluor objectives for imaging (Numerical Aperature 0.3 and 0.5, respectively). Images were transferred to ImageJ software was used to quantify the number of cFos positive GnRH neurons and KP-positive neurons within defined regions. For cFos positive GnRH neurons and total quantification of GnRH neurons, sections were quantified on coded slides manually.

### LH ELISA assay

LH levels were determined by a sandwich ELISA as described previously^41^ using the mouse LH–RP reference provided by A. F. Parlow (National Hormone and Pituitary Program, Torrance, CA). For all experiments, animals were habituated for 3-weeks prior to experiments, and on the day of experiments, blood was harvested in 10-min intervals (4 ul/sample) over a 2-hr period for each sex and genotype in PBS-Tween (0,05%).

### qRT-PCR

Adult *nes::cre;Dmxl2*^*wt/loxp*^ and *Dmxl2*^*wt/loxp*^ mice (*n* = 5–10 of each genotype and sex) were anesthetized with isoflurane vapor and immediately decapitated. After hypothalamic dissection, mRNA was extracted using Trizol (Invitrogen, Carlsbad CA, USA), as previously described^9^. 1 μg of total RNA was used for the synthesis of OligoDT cDNA from the Superscript III First-Strand cDNA Synthesis kit (Invitrogen), following the manufacturer’s instructions. 2 μl of 1/50 diluted hypothalamic cDNA in duplicates was used for real time quantitative PCR (RT-qPCR) using SyberGreen MasterMix (Bio-Rad, Hercules, CA). Samples were processed in an ICycler qRT-PCR machine (Bio-Rad). Primer sequences for *Kiss1* were 5′*-*TAACGAGTTCCTGGGGTCCG*-*3′; 5′*-*CTCCTGCTTCTCCTCTGTGT*-*3′*. GAPDH* was used as an internal control (5′*-GATGCCTGCTTCACCACCTTCT-*3′; 5′*-AATGTGTCCGTCGTGGATCTGA-*3′). All primers were used at a concentration of 0.15 μM. qRT-PCR conditions leading to an efficiency between 95–110% were selected. Relative differences in the cDNA concentration between baseline and experimental conditions were calculated using the comparative threshold cycle (Ct) method.

### Statistical Analysis

Statistical analyses were carried out using Prism software (GraphPad, La Jolla, CA). Comparisons between two groups were analyzed with a non-parametric Mann Whitney unpaired t-test. For multiple treatments and comparisons, one-way Anova and a post-hoc Newman-Keuls test were performed. Differences were considered significant when *p* < 0.05. All data are expressed as mean ± SEM for each group, genotype, and experiment.

## Additional Information

**How to cite this article:** Tata, B. K. *et al*. Rabconnectin-3α is required for the morphological maturation of GnRH neurons and kisspeptin responsiveness. *Sci. Rep.* **7**, 42463; doi: 10.1038/srep42463 (2017).

**Publisher's note:** Springer Nature remains neutral with regard to jurisdictional claims in published maps and institutional affiliations.

## Supplementary Material

Supplementary Table S1
