# Supplementary material for: Rabconnectin-3α is required for the morphological maturation of GnRH neurons and kisspeptin responsiveness
Source: Sci Rep. 2017 Feb 17;7:42463. doi: 10.1038/srep42463 (PMC5314327; doi:10.1038/srep42463)
Supplement: Supplementary Table S1 [file srep42463-s1.doc]

**Rabconnectin-3α is required for the morphological maturation of GnRH neurons and kisspeptin responsiveness**

Brooke K. Tata ^1^, Carole Harbulot ^1^, Zsolt Csaba ^1^, Stéphane Peineau ^1,2^, Sandrine Jacquier ^1^, Nicolas de Roux ^1,3,*^.

**Affiliations**:

^1^ Univ Paris Diderot, Sorbonne Paris Cité, U1141, Inserm, F- 75019, Paris, France.

^2^ MRC Centre for Synaptic Plasticity; School of Physiology, Pharmacology, Neurosciences; University of Bristol; Bristol, UK

^3^ AP-HP, Laboratoire de Biochimie. Hôpital Robert Debré, Paris, F-75019, France.

**Table S1. List of primary antibodies**

| **Primary antibody** | **Host species** | **Source** | **Catalogue number** | **Concentration** |
| --- | --- | --- | --- | --- |
| Polyclonal anti **DMXL2** | Rabbit | Sigma | HPA039375 | 1/500 |
| Polyclonal **anti-kisspeptin** (mammalian) | Rabbit | Dr. Alain Caraty (INRA, Tours, France) | AC 564 | 1/10000 |
| Polyclonal **anti-GnRH** | Guinea Pig | Dr. Greg Anderson (CNE, University of Otago) | GA04 | 1/15000 |
| Polyclonal **anti-cFos** | Rabbit | Santa Cruz Biotechnology | SC-52 | 1/5000 |
